# Query-Informed Multi-Agent Motion Prediction

**DOI:** 10.3390/s24010009

**Published:** 2023-12-19

**Authors:** Chong Guo, Shouyi Fan, Chaoyi Chen, Wenbo Zhao, Jiawei Wang, Yao Zhang, Yanhong Chen

**Affiliations:** 1 College of Automotive Engineering, Jilin University, Changchun 130025, China; guochong@jlu.edu.cn (C.G.); syfan22@mails.jlu.edu.cn (S.F.); chency21@mails.jlu.edu.cn (C.C.); wjw20@mails.jlu.edu.cn (J.W.); zyao18@mails.jlu.edu.cn (Y.Z.); 2 Changsha Automobile Innovation Research Institute, Changsha 410005, China; 3 FAW Car Co., Ltd., Changchun 130015, China; zhaowenbo@fawcar.com.cn

**Keywords:** autonomous vehicles, trajectory prediction, query-informed, multimodal

## Abstract

In a dynamic environment, autonomous driving vehicles require accurate decision-making and trajectory planning. To achieve this, autonomous vehicles need to understand their surrounding environment and predict the behavior and future trajectories of other traffic participants. In recent years, vectorization methods have dominated the field of motion prediction due to their ability to capture complex interactions in traffic scenes. However, existing research using vectorization methods for scene encoding often overlooks important physical information about vehicles, such as speed and heading angle, relying solely on displacement to represent the physical attributes of agents. This approach is insufficient for accurate trajectory prediction models. Additionally, agents’ future trajectories can be diverse, such as proceeding straight or making left or right turns at intersections. Therefore, the output of trajectory prediction models should be multimodal to account for these variations. Existing research has used multiple regression heads to output future trajectories and confidence, but the results have been suboptimal. To address these issues, we propose QINET, a method for accurate multimodal trajectory prediction for all agents in a scene. In the scene encoding part, we enhance the feature attributes of agent vehicles to better represent the physical information of agents in the scene. Our scene representation also possesses rotational and spatial invariance. In the decoder part, we use cross-attention and induce the generation of multimodal future trajectories by employing a self-learned query matrix. Experimental results demonstrate that QINET achieves state-of-the-art performance on the Argoverse motion prediction benchmark and is capable of fast multimodal trajectory prediction for multiple agents.

## 1. Introduction

Multi-object motion prediction is an essential step in autonomous driving. It aids autonomous vehicles in making informed decisions and prevents accidents. However, traffic scenes are highly complex, involving targets, road networks, and their interactions. Prediction models need to take these entities as inputs and output reasonable multimodal trajectories that intelligent agents may take in the future.

Recently, deep learning-based methods have shown promising results in motion prediction tasks [1,2,3,4]. Some studies rasterize scenes into top–down views and employ CNNs for prediction [1,5,6]. While these methods are easily implementable with off-the-shelf image models, they have limited applicability and come with a high cost. Given these constraints, recent research [2,4] has adopted a vectorization approach for a more efficient scene representation, extracting a set of vector nodes from the trajectories of traffic participants and map elements. Subsequently, to learn relationships between vectorized entities, such as trajectory waypoints and lane segments, some studies [7,8,9] use graph neural networks to process scenes, some studies [10] use transformers to process scenes, others [11] use point cloud models to process scenes. In addition, some research [12,13,14] has focused on the vulnerability of data-driven algorithms, Autoencoder, and GAN for trajectory prediction.

However, during the scene encoding process, the perspective of predicting the scene varies for each target. Most existing methods transform the entire scene with respect to one target agent at a time, which leads to asymmetry for other agents. This approach has lower prediction efficiency and is less robust to co-ordinate transformations. To address these issues, HiVT [15] uses rotation invariant and shift invariant scene representations, which greatly enhances the robustness of the prediction model. Additionally, many studies simply use displacement to encode vectors without considering other physical information of scene entities, such as speed, heading angle, and spatial relationships between entities. This oversight may lead to suboptimal trajectory predictions. In the decoder section, the predicted trajectories should be multimodal. However, current research [16] mostly employs non-maximum suppression (NMS) methods, setting a certain threshold, and filtering out multimodal trajectories based on L2 distances between future trajectory endpoints, which yields unsatisfactory results.

We employ a symmetric scene representation using HiVT, in which all relative features possess translational and rotational invariance. Building upon this, we introduce a query-based framework for inducing the generation of multimodal trajectories: QINET. In terms of scene encoding, our representation method incorporates additional relevant features about the target and lane segments to convey their physical significance. In the trajectory prediction decoder section, to enhance the multimodality of output trajectories, we introduce self-learnable parameter matrices for cross-attention over the agent vehicle’s history, which we refer to as the query mechanism.

QINET consists of an encoder module and a decoder module. In the encoder section, we expand the scene representation of vectorized entities, incorporating new features related to targets and lane segments. We use projected vector representations for all relevant features of the traffic scene at each timestamp, providing a more detailed description of relative spatial relationships. In the decoder section, we develop a query-informed architecture that combines features from agent history, agent relationships, and the road graph as a target-centric scene representation. This is achieved through querying and self-attention learning to extract and combine scene features. The combined feature representation is used to induce the output of multimodal trajectories. Some feature representation comes from the agent’s historical trajectory features, while another part comes from the output of the encoder section, which we refer to as anchor points. In this way, the network first learns to generate scene modal features with maximum diversity without environmental constraints, and then decodes future trajectories after absorbing anchor points carrying rich target-oriented contextual information.

Our contributions can be summarized as follows: first, we extend HiVT’s scene representation method by incorporating new features related to targets and lane segments, providing a more detailed description of relative spatial relationships; second, we propose a queries-informed decoder based on DETR [17], which combines historical query information and anchor point information end-to-end, promoting the multimodality of output trajectories; and, third, the designed QINET is capable of making accurate and reasonable predictions.

## 2. Related Work

Traffic Scene Representation. Solving the motion prediction problem requires learning rich representations from elements in the traffic scene, including high-definition maps and the agent’s past trajectories. A significant amount of research employs grid-based scene representations as model inputs [18,19,20] and utilizes standard image models [21,22] for learning. Specifically, these methods extract map elements (such as lane boundaries, stop lines, and crosswalks) from high-definition maps and render the scene into a top–down image using different colors. The agent’s past trajectories are either rasterized into additional image channels [1,5] or processed by temporal models like RNNs [23,24].

Rasterization methods require mature computer technology support, but their drawback lies in being inefficient and costly. Recently, vectorization methods [2,4,25] have gained popularity due to their efficient sparse encoding and ability to capture complex structural information. Unlike rasterization methods, these approaches consider the scene as a set of entities associated with semantic and geometric attributes, and learn the relationships between these entities. VectorNet [25] employs graph neural networks to model interactions between lanes and trajectory polylines. It has also served as a backbone in some subsequent works [26,27]. LaneGCN [2] constructs lane graphs from lane segments and employs multi-scale graph convolutional networks to capture long-range dependencies by learning features of graph nodes. TPCN [4] extends point cloud models to learn from a spatial-temporal set composed of trajectory waypoints and lane points. Our scene representation falls under this category as well, but what sets it apart is that all vectorized entities are characterized by relative positions, enhancing model robustness as relative displacements remain invariant to translation. The approach most related to this paper is HiVT [15], which uses a translation-invariant scene representation method that avoids using absolute positions and characterizes the geometric entities with relative positions and constructs rotation-invariant transformers to model the different interactions between the vectorized entities locally and globally.

Motion Prediction. Since social interactions are ubiquitous in traffic scenes and significantly influence the future motions of traffic agents, many motion prediction methods consider dependencies between agent behaviors and rational agent–agent interactions. They employ social pooling [28,29], graph neural networks [30,31], or attention mechanisms [20,25,32,33,34]. Inspired by the success of transformer models [10] in various fields, some recent works utilize transformers in motion prediction tasks to model spatial relationships, temporal dependencies, and relationships between agents and map elements [23,32,34,35].

In contrast, our transformer architecture differs from existing architectures by incorporating hierarchical learning of local and global representations. We encode each timestep in the local encoder, breaking down the time. In addition, we decompose the space by modeling multiple agents with a goal-centered representation that is invariant to translation and rotation in the scene. In the global encoder, we interact with all cars in the scene to obtain remote dependency information. The combination of a hierarchical structure and symmetric design allows our approach to achieve state-of-the-art prediction performance with fewer parameters and lower computational costs.

## 3. Method

### 3.1. Overall Framework

**Figure 1:**
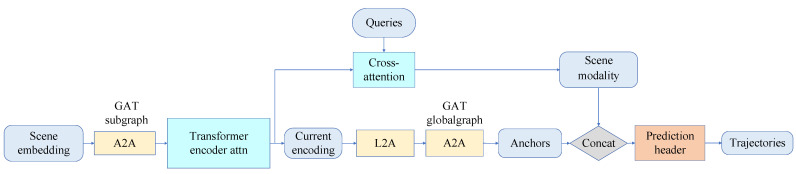
This is the overall framework of QINET. We utilize the graph attention mechanism (GAT) to establish A2A and L2A for extracting environmental features around participants. We set query matrices (queries) to query the historical trajectory features of the agent, obtaining diverse scene-modal features. These scene-modal features, combined with anchor features learned from the global graph output, are used to output multimodal trajectories.

This section proposes a query-informed vehicle trajectory prediction method, QINET, to induce the model to generate multi-modal predicted trajectory to the maximum extent. The overall process is shown in Figure 1. The prediction model consists of three parts: scene vectorization representation, encoder, and decoder. Firstly, in the scene vectorization representation part, we extend the feature representation in HiVT [15]. For the vector node representation of agent, we use the displacement of agent and its absolute value, velocity, and its absolute value, sine–cosine value of heading angle, and timestamp length information. For lane vector node representation, we use lane segment displacement and heading angle to represent. Such scenarios represent a more detailed description of the relative spatial relationships between vector nodes. Then, our feature extraction encoder makes use of HiVT’s encoder architecture to design subgraphs for local encoding. Transformer encoder is used to pay attention to time information, and global graph is used to extract and interact remote dependent information. In addition, we propose a query-informed multi-scene modality for an end-to-end learning approach to induce output multimodal prediction trajectories. In our method, the proposed generation is obtained by querying the output current encoding of transformer encoder by cross-attention method, which is the information extraction of agent historical trajectory. The multi-modality of the predicted trajectory can be promoted to the maximum extent without environmental constraints. In addition, we obtain the anchor feature representation that absorbs and carries rich goal-oriented context information through self-attentional learning of global graph output. A portion of the input to the multi-modal trajectory prediction decoder comes from the query-informed multi-scene modality features, while another portion comes from anchor features.

### 3.2. Complexity Analysis

In the local encoder part, we utilized the model architecture of HiVT to decompose time and spatial dimensions, learning spatial relationships locally at each timestep. This approach reduces complexity from $O\left({\left(NT+L\right)}^{2}\right)$ to $O\left(NT^{2}+TN^{2}+NL\right)$, where *N*, *T,* and *L* represent the number of agents, historical timesteps, and the number of lane segments, respectively. Although we expanded the feature dimension of nodes in HiVT, this expansion has minimal impact on the overall complexity. In addition, in the decoder part, the complexity added by the method of designing query matrices to obtain multimodal scene feature representations can be calculated as $O\left(NT\right)$, which is lightweight compared to the local encoder.

### 3.3. Scene Representation

#### 3.3.1. Node Feature Representation

For the traffic agent, we extract trajectory segments at each timestamp, which take the form of directed splines. These trajectory segments, referred to as vector nodes, are characterized by their feature attributes as: $n_{a}=\left\{R_{i}^{T^{⊤}}d_{i}^{t},R_{i}^{T^{⊤}}v_{i}^{t},R_{i}^{T^{⊤}}a_{i}^{t},\Delta t^{t},b_{i}|i=1,\ldots ,N_{t},t=0,\ldots ,19\}\right.$. The main feature attributes are highlighted in red as follows:

(1) $$l_{i}^{t}=\left[x_{i}^{t},y_{i}^{t}\right]$$

(2) $$d_{i}^{t}=l_{i}^{t}-l_{i}^{t-1}$$

(3) $$d_{x_{i}}^{t}=x_{i}^{t}-x_{i}^{t-1}$$

(4) $$d_{y_{i}}^{t}=y_{i}^{t}-y_{i}^{t-1}$$

(5) $$v_{i}^{t}=\frac{d_{i}^{t}}{\Delta t^{t}}$$

(6) $$\alpha_{i}^{t}=\;\arctan\frac{d_{y_{i}}^{t}}{d_{x_{i}}^{t}}.$$

(7) $$a_{i}^{t}=\left[\cos\left(\alpha_{i}^{t}\right),\sin\left(\alpha_{i}^{t}\right)\right]$$

(8) $$\Delta t^{t}=t^{t}-t^{t-1}$$

where $N_{t}$ represents the total number of agent vehicles appearing at timestamp *t*. $l_{i}^{t}$ denotes the co-ordinates of the *i*-th agent in the scene at timestamp *t*. $d_{i}^{t}$ is the displacement vector of agent vehicle *i* from timestamp *t* − 1 to *t*. $v_{i}^{t}$ represents the speed. $\alpha_{i}^{t}$ indicates the heading angle of the i-th agent at timestamp *t*. $a_{i}^{t}$ is the heading vector composed of the cosine and sine of the agent’s heading angle. $\Delta t^{t}$ represents the duration of the timestamp. Including this in the node features is considered due to the non-uniform sampling frequency of the Argoverse dataset, which is not consistently 0.1 s. $R_{i}^{T}$ is the rotation matrix defined by the heading angle of the *i*-th agent at the current timestep (*t* = 19). $b_{i}$ represents the semantic feature.

For lane vector nodes, we opt to extract the co-ordinates of lane points along with their associated semantic attributes, such as dashed or solid lines, and turning directions. We vectorize lane segments into nodes similar to agent vectors, and represent them as $n_{l}=\left\{d_{k},a_{k},b_{k}|k=1,\ldots ,N_{l}\right\}$, where $N_{l}$ denotes the total number of lane segments, $d_{k}$ represents the displacement vector of the lane segment, $a_{k}$ is composed of the sine and cosine values of the heading angle of the lane segment, indicating the direction of the displacement vector, and $b_{k}$ denotes the semantic attribute. The specific expressions for $d_{k}$ and $a_{k}$ are as follows:

(9) $$d_{k}=l_{k}^{1}-l_{k}^{0}$$

(10) $$a_{k}=\left[\cos\left(\alpha_{k}\right),\sin\left(\alpha_{k}\right)\right]$$

where $l_{k}^{1}$ and $l_{k}^{0}$ represent the endpoint and starting point of the lane segment, respectively. In the vectorized node representation, we abstain from using any absolute positions and instead utilize relative positions. This ensures that the node feature attributes possess translational invariance.

#### 3.3.2. Edge Feature Representation

Node features only represent the characteristics of agent vehicles and lane segments. The graph attention mechanism requires specifying attention targets in the scene, and encoding the features of edges between the target agent vehicle and the attention targets. Therefore, we introduce attributes for the edges between entities. For the edge attributes between agents, we describe them as follows: $e_{aa}=\left\{R_{i}^{T}d_{ij}^{t},R_{i}^{T}v_{ij}^{t},d_{j2i}^{t},v_{j2i}^{t},a_{j2i}^{t}|t=0,\ldots ,19;i,j=1,\;i\neq j\}\right.$, the details are as follows:

(11) $$d_{ij}^{t}=l_{j}^{t}-l_{i}^{t}$$

(12) $$v_{ij}^{t}=v_{j}^{t}-v_{i}^{t}$$

(13) $$\alpha_{ij}^{t}=\alpha_{j}^{t}-\alpha_{i}^{t}$$

(14) $$a_{j2i}^{t}=\left[\cos\left(\alpha_{ji}^{t}\right),\sin\left(\alpha_{ji}^{t}\right)\right]$$

(15) $$d_{j2i}^{t}=R_{i}^{t^{⊤}}d_{ji}^{t}=\left[d_{j2ix}^{t},d_{j2iy}^{t}\right]$$

(16) $$v_{j2i}^{t}=R_{i}^{t^{⊤}}v_{ji}^{t}=\left[v_{j2ix}^{t},v_{j2iy}^{t}\right]$$

where $d_{ij}^{t}$ is the relative displacement vector between agent *i* and agent *j*, $v_{ij}^{t}$ is the velocity vector between them, $R_{i}^{t}$ is the rotation matrix parameterized by the heading angle of center agent *i* at timestamp *t*, $a_{j2i}^{t}$ represents the relative heading angle vector, $d_{j2i}^{t}$ expresses the lateral and longitudinal distance of agent *j* relative to agent *i* at timestamp *t*, and $v_{j2i}^{t}$ represents the lateral and longitudinal velocity of agent *j* relative to agent *i* at timestamp *t*, where *x* denotes lateral relative to the center node agent *i*, and *y* denotes longitudinal relative to the center node agent *i*.

For the edge attributes between agent nodes and lane nodes, we describe them as follows: $e_{al}=\left\{R_{i}^{T}d_{ik}^{t},d_{i2k}^{t},v_{i2k}^{t},a_{i2k}^{t}|t=\left.0,\ldots ,19;i=1,\ldots ,N_{t};k==1,\ldots ,N_{l}\right\}\right.$; the details are as follows:

(17) $$d_{ik}^{t}=l_{k}^{0}-l_{i}^{t}$$

(18) $$d_{i2k}^{t}=R_{k}^{t^{⊤}}d_{ik}^{t}=\left[d_{i2kx}^{t},d_{i2ky}^{t}\right]$$

(19) $$v_{i2k}^{t}=R_{k}^{t^{⊤}}v_{i}^{t}=\left[v_{i2kx}^{t},v_{i2ky}^{t}\right]$$

(20) $$\alpha_{ik}^{t}=\alpha_{k}^{t}-\alpha_{i}^{t}$$

(21) $$a_{i2k}^{t}=\left[\cos\left(\alpha_{ik}^{t}\right),\sin\left(\alpha_{ik}^{t}\right)\right]$$

where $d_{ik}^{t}$ is the relative position vector between agent *I* and lane segment *k* at timestamp *t*, $R_{k}^{t}$ is the rotation matrix parameterized by the heading angle $\alpha_{k}^{t}$ of lane node *k*, $d_{i2k}^{t}$ represents the lateral and longitudinal distance from agent *i* to lane segment *k*, where *x* is lateral relative to lane segment *k* and *y* is longitudinal relative to lane segment *k*, $v_{i2k}^{t}$ represents the relative velocity vector, $v_{i2kx}^{t}$ and $v_{i2ky}^{t}$, respectively, denote the lateral and longitudinal velocity of agent *i* relative to lane segment *k*, and $a_{i2k}^{t}$ is the relative heading angle vector.

The relative positions and velocities we propose describe the distance between two independent nodes, as well as the speed at which one node moves laterally and longitudinally towards another node. Compared to absolute representations, this relative representation provides a more detailed description of the interactions between entities, allowing downstream networks to better understand their behaviors. Additionally, this lateral and longitudinal relative representation naturally ensures translational and rotational invariance.

### 3.4. Encoder

#### 3.4.1. Local Encoder

**Figure 2:**
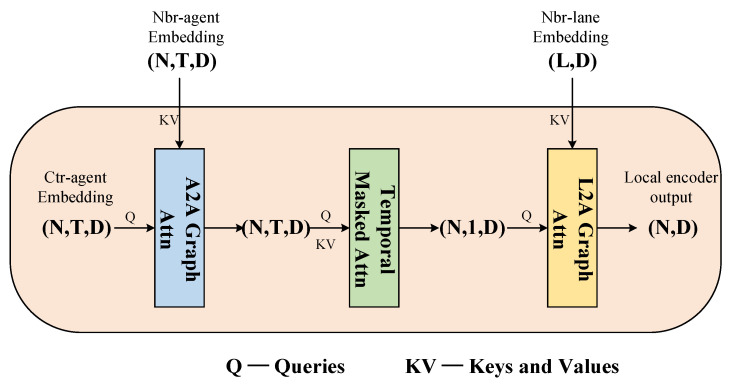
Overview of the local encoder diagram: the query always originates from the central agent node feature. For A2A, we extract key and value from neighboring agent nodes. Self-attention is employed in the temporal encoder. For L2A, key and value are sourced from neighboring lane nodes. In the diagram, N represents the number of agents in the scene, T denotes the number of timesteps, and L represents the number of lane nodes.

The local encoder processes the temporal scene graph in two stages, as shown in Figure 2. In the first stage, it models the agent–agent interactions for each timestep, which we refer to as A2A. For A2A, we perform local interactions centered around each agent within a limited range. After the interaction between entities, we use a time transformer encoder module to capture temporal dependencies across the traffic scene. In the second stage, we extract the features from the last timestep of the output of the transformer encoder. These features contain information about the central agent’s vehicle at the current timestep, as well as interaction information with nearby other agent vehicles in both spatial and temporal dimensions. We use these features for modeling lane–agent interactions, which we refer to as L2A. With this, after the local encoder is completed, we obtain agent features that are enriched with rich contextual information.

Agent–Agent Interaction. During the A2A step, we utilize a graph neural network. We employ multi-head cross-attention to understand the influence of different surrounding agent vehicles within each local range on the central agent vehicle. Specifically, we first apply multi-layer perceptions (MLPs) to the node attributes of the central agent vehicle and the corresponding edge attributes. This allows us to obtain a time-variant encoding $Z_{i}=\{z_{i}^{t}|t=1,\ldots ,T\}$ for the central agent node *i*, along with time-variant encodings for the surrounding neighboring nodes associated with it:

(22) $$\left.z_{i}^{t}=\phi_{\mathrm{center}\;}\left(R_{i}^{T^{⊤}}d_{i}^{t},R_{i}^{T^{⊤}}v_{i}^{t},R_{i}^{T^{⊤}}a_{i}^{⊤},\Delta t^{t},b_{i}\right]\right)$$

(23) $$z_{ij}^{t}=\phi_{\mathrm{nbr}}\left(\left[R_{i}^{T^{⊤}}d_{j}^{t},R_{i}^{T^{⊤}}d_{ij}^{t},R_{i}^{T^{⊤}}v_{ij}^{t},d_{j2i}^{t},v_{j2i}^{t},a_{j2i}^{t},b_{j}\right]\right)$$

where $\phi_{\mathrm{center}\;}$ and $\phi_{\mathrm{nbr}}$ represent MLP modules. Due to the use of relative vectors and the presence of rotation matrices, both the node attributes of the central node and its associated edge attributes possess translational and rotational invariance. Next, we use cross-attention to fuse the central node features and its edge features. The query part of the cross-attention is derived from the central node attribute $z_{i}^{t}$, while the key and value parts come from the edge attributes $z_{ij}^{t}$. Subsequently, we perform dot product [10] and gating operations [15], resulting in the output ${\hat{Z}}_{i}=\{{\hat{z}}_{i}^{t}|t=1,\ldots ,T\}$. We then further apply an MLP to ${\hat{Z}}_{i}$ and use residual connections to obtain the merged feature encoding $S_{i}=\{s_{i}^{t}|t=1,\ldots ,T\}$, which contains information about agent interactions and updates after the interaction.

Temporal Dependency. To further capture temporal dependencies, we apply a transformer encoder to the output $S_{i}$ of the A2A step. Following the approach of BERT [36], we introduce learnable position embeddings at each timestamp and stack them onto $S_{i}$ to obtain the new matrix ${\hat{S}}_{i}\in {\mathbb{R}}^{T\times d_{h}}$. Unlike previous studies [15], we do not add an extra learnable token at the end position, resulting in ${\hat{S}}_{i}\in {\mathbb{R}}^{\left(T+1\right)\times d_{h}}$. Instead, we directly process ${\hat{S}}_{i}$ through the transformer encoder to obtain the updated sequence features $H_{i}=\left\{h_{i}^{t}|t=1,\ldots ,T\right\}$ and extract the final node feature $h_{i}^{T}$ belonging to the current timestep. This feature is then fed into the subsequent L2A module, as we have observed improved performance with this approach. During the transformer encoding process, a time mask is applied to enforce tokens to only attend to preceding timesteps.

Agent–Lane Interaction. To facilitate information interaction between agents and lane segments, we apply another multi-head cross-attention module. First, we use a multi-layer perceptron to encode the edge features between the central agent node *i* and nearby lane nodes:

(24) $$z_{ik}=\phi_{\mathrm{lane}\;}\left(\left[R_{i}^{T^{⊤}}d_{k},R_{i}^{T^{⊤}}d_{ik},d_{i2k},v_{i2k},a_{i2k},b_{k}\right]\right)$$

where $\phi_{\mathrm{lane}\;}$ represents an MLP module. We use the current timestep’s agent node feature $h_{i}^{T}$ from the transformer encoder output as the query, and the edge attributes $z_{ik}$ between the agent and the lane segment as the key and value. The field of view is an adjustable threshold used to limit the lane nodes that need to be interactively fused with the central agent node. We obtain the final node embedding $h_{i}$ for central agent *i*. It encapsulates a rich spatiotemporal representation fused by agent *i*, combining the dynamic characteristics of agent *i* with its iterative interactions with the surrounding environment. The final local representation for all agents is defined as $H=\left\{h_{i}|i=1,\ldots ,N\right\}$, where *N* is the number of agents.

#### 3.4.2. Global Encoder

The local encoder only achieves information interaction within a local scope, lacking remote dependency relationships within the scene. Therefore, we designed a global encoder. Similar to the A2A module in the local encoder, we employ an MLP to encode the edge attributes between agent *i* and agent *j*.

(25) $$g_{ij}=\phi_{\mathrm{rel}}\left(\left[R_{i}^{T^{⊤}}d_{ij}^{T},R_{i}^{T^{⊤}}v_{ij}^{T},d_{j2i}^{T},v_{j2i}^{T},a_{j2i}^{T}\right]\right)$$

Here, T represents the current timestep. Afterwards, we use $h_{i}$ as the query, $\left[h_{j},g_{ij}\right]$ as the key and value:

(26) $$q_{i}=W^{Q^{\mathrm{global}\;}}h_{i},$$

(27) $$k_{ij}=W^{K^{\mathrm{global}\;}}\left[h_{j},g_{ij}\right],$$

(28) $$v_{ij}=W^{V^{\mathrm{global}\;}}\left[h_{j},g_{ij}\right],$$

where $W^{Q^{\mathrm{global}}}$, $W^{K^{\mathrm{global}}}$, and $W^{V^{\mathrm{global}}}$ are linear transformation matrices. Then, we apply a multi-head cross-attention module to update the features of agent i:

(29) $$\alpha_{i}=\mathrm{softmax}\left(\frac{q_{i}^{T}}{\sqrt{d_{k}}}\cdot \left[{\left\{k_{ij}\right\}}_{j\in N_{i}}\right]\right)$$

(30) $${\hat{h}}_{i}=\underset{j\in N_{i}}{\sum }\alpha_{ij}v_{ij}$$

where $N_{i}$ contains the neighboring agents that central agent *i* needs to interact with, $\alpha_{ij}$ represents the score weight of neighbor agent *j* relative to agent *i*. Furthermore, we pass the updated neighbor agent feature ${\hat{h}}_{i}$ and the central agent feature $h_{i}$ through a gating step [15] before inputting them into the MLP module to obtain the output of the global graph $\tilde{h_{i}}$, with feature dimensions denoted as [*K*, *N*, and *D*]. Here, *K* is the number of heads in the multi-head cross-attention module, representing the number of modes in the output trajectory, *N* denotes the number of agents, and *D* is the feature dimension.

### 3.5. Decoder

#### 3.5.1. Query-Informed Multi-Scene Modality Creation

**Figure 3:**
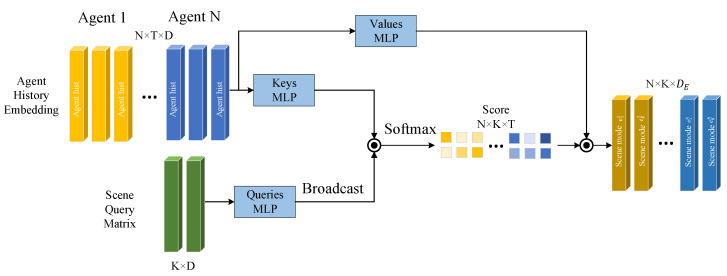
Construction diagram of multi-scene modality. We establish a learnable scene query matrix to query the historical trajectory features of agents, obtaining modality features for multiple scenes for decoding future trajectories.

As shown in Figure 3, we relinquish the constraints of specific driving scenarios and aim to maximize the diversity of future trajectory candidates by first creating multi-modal scenes through querying from the motion history of the target agent. Specifically, inspired by the approach of setting object queries in DETR [17], we define a set of learnable parameters forming a query matrix $Q_{\mathrm{scene}\;}\in {\mathbb{R}}^{K\times D}$ to attend to the output $H_{i}=\{h_{i}^{t}|t=1,\ldots T\}\in {\mathbb{R}}^{T\times D}$ from the temporal dependency module. This is achieved by generating *K* scene modal features $E_{\mathrm{scene}}=\left\{E_{\mathrm{scene}}^{k}|k=1,\ldots ,K\right\}\in {\mathbb{R}}^{K\times D_{E}}$ through a cross-attention mechanism:

(31) $$E_{\mathrm{scene}}=Softmax\left(\frac{\left(Q_{scene}W_{q}\right){\left(H_{i}W_{k}\right)}^{T}}{\sqrt{D_{E}}}\right)\left(H_{i}W_{v}\right),$$

(32) $$score_{k}=softmax\left(\alpha_{k}\right)$$

(33) $$E_{scene}^{k}=\underset{t=1,\ldots ,T}{\sum }score_{kt}(W_{q}^{T}h_{i}^{t})$$

where $W_{q}$, $W_{k}$, and $W_{v}\in R^{D\times D_{E}}$ are linear transformation matrices. $\alpha_{kt}$ represents the contribution of the agent’s feature at the *t*-th historical timestamp to the *k*-th scene mode. We apply a softmax operation to the contributions of all timestamps corresponding to the *k*-th scene mode to obtain the vector $score_{k}$. The contribution scores corresponding to each timestamp in $score_{k}$ are multiplied with the feature of the agent at that timestamp and then summed, resulting in the queried *k*-th scene modal feature. Since the scene features are derived from the agent’s historical trajectory encoding and do not contain semantic features of the surrounding environment, this ensures maximum multimodality of the scene. $D_{E}$ represents the dimension after linear transformation. The denominator $\sqrt{D_{E}}$ is used for normalization and to prevent the dot product from becoming too large, which might lead to saturation in the softmax operation.

#### 3.5.2. Anchor Learning

The anchors are learned end-to-end in the network to convey target-oriented environmental information while preserving diversity. We set the number of anchors to *K*, which is equal to the number of environmental modes, so that each anchor corresponds to one scene mode. We apply an MLP to the output of the global graph $\tilde{h_{i}}$ to generate anchor features corresponding to each scene mode:

(34) $$E_{\mathrm{anchor}}=\phi_{\mathrm{anchor}}\left(\tilde{h_{i}}\right)$$

where $\phi_{\mathrm{scene}}$ represents an MLP module. Our model does not directly utilize predicted endpoints, but rather leverages their embeddings (i.e., pre-output features) as anchor points $E_{\mathrm{anch}\;}=\left\{E_{\mathrm{anch}}^{i}|i=1,\ldots ,K\right\}$. These anchor points are used to inject target-oriented scene context into the generated multimodal scene features $E_{\mathrm{scene}\;}$. This is a new type of approach compared to previous research. In TNT [16], anchor points are manually sampled uniformly from the map. In MultiPath [1], anchor points are predefined trajectories clustered from training data. In MultiPath++ [37], anchor points are learnable model parameters that are fixed after training and independent of the input. In contrast, we propose using anchor embeddings to facilitate trajectory learning. Compared to TNT and MultiPath, our anchors are more adaptive and convenient to obtain through end-to-end learning. Compared to MultiPath++, our anchors correspond to individual samples, thus carrying specific sample-specific information.

#### 3.5.3. Trajectory Prediction Head

As described above, the multimodal scene encoding $E_{\mathrm{scene}\;}$ can be seen as unconstrained future trajectories inferred solely from the agent’s history, while anchors $E_{\mathrm{anchor}}$ convey target-based contextual information. Here, we combine both to allow the network to make further selections and refinements:

(35) $$E_{\mathrm{final}\;}=W_{1}E_{\mathrm{scene}}+W_{2}E_{\mathrm{anchor}\;}$$

where $W_{1}$ and $W_{2}$ are linear transformation matrices. Taking the fused features as input, the multimodal prediction head outputs the final motion predictions. For each participant, it predicts *K* possible future trajectories along with their confidences. The head has two branches: one regression branch predicting the trajectories for each mode, and one classification branch predicting the confidence scores for each mode. For the *i*-th participant, we apply residual blocks and linear layers to regress the *K* sequences of relative co-ordinates in the regression branch:

(36) $$O_{i,\;\mathrm{reg}\;}={\left\{\left(p_{i,1}^{k},p_{i,2}^{k},\ldots ,p_{i,T}^{k}\right)\right\}}_{k\in \left[0,K-1\right]}$$

where, $p_{i,t}^{k}$ represents the predicted relative co-ordinates of the *i*-th participant in the *k*-th mode at timestep *t*, i.e., co-ordinates in the local co-ordinate system with the historical endpoint of the *i*-th participant as the origin. For the classification branch, we apply an MLP to $p_{i,T}^{k}-p_{i,0}$ to obtain *K* distance embeddings, where $p_{i,0}$ is the last point of the historical trajectory of the *i*-th agent. Then, we concatenate each distance embedding with the agent features, apply residual blocks and linear layers to output *K* confidence scores, $O_{i,\mathrm{cls}}=\left(c_{i,0},c_{i,1},\ldots ,c_{i,K-1}\right)$.

## 4. Simulation Results

### 4.1. Experimental Settings

#### 4.1.1. Dataset

We utilize the Argoverse motion forecasting dataset [38], which comprises real-world traffic scenarios with agent trajectories and high-definition maps. The dataset encompasses 324,557 authentic traffic scenes. The training, validation, and test sets include 205,942, 39,472, and 78,143 scenes, respectively. Each scene is a 5 s sequence sampled at 10 Hz, containing the positions of all agents in the past 2 s. In the Argoverse motion forecasting challenge, the task is to predict the future positions of a target agent for the next 3 s based on an initial observation of the first 2 s of the scene.

#### 4.1.2. Metrics

We adhere to the Argoverse benchmark and evaluate our model using metrics including minimum average displacement error (minADE), minimum final displacement error (minFDE), and miss rate (MR). These metrics allow the model to predict up to 6 trajectories for each agent.

#### 4.1.3. Implementation Details

We trained all models using the AdamW optimizer [39] with an initial learning rate of 0.001 for 64 epochs. We employed a cosine annealing scheduler for learning rate decay. The number of layers for the agent–agent transformer, agent–lane transformer, temporal transformer, and global encoder was set to 1, 1, 4, and 3, respectively. The number of hidden units was 128, and there were 8 heads in all multi-head attention blocks. The local region radius for A2A was set to 20 m, and for L2A it was set to 50 m. We did not predict agents that appeared for less than two steps, unless it was the target agent.

#### 4.1.4. Comparison with State-of-the-Art

In Table 1, we present the results of QINET on the Argoverse motion prediction test set, comparing it with other state-of-the-art models. The data in Table 1 are sourced from the Argoverse leaderboard. QINET outperforms all other methods in terms of minADE and minFDE, and maintains a competitive ranking in MR, verifying the superior predictive performance of our method. The sacrifice in the MR metric stems from the decoder’s multimodal influence on the generated trajectories, but this influence improves the accuracy of trajectory prediction in some scenarios.

In the validation set section, we compared our results with HiVT. We found that before model ensemble, our model performs better on the Argoverse validation set compared to HIVT, as shown in Table 2 with specific metrics.

#### 4.1.5. Ablation Studies

Our ablation study consists of four parts: the importance of each module in QINET, the importance of expanded scene representation, and the importance of layered lane transformers. We conducted these experiments on the Argoverse validation set.

Importance of Each Module.

To investigate the importance of each module for the overall network, we individually removed each module and tested its contribution on the Argoverse test set, as shown in Table 3. Each module contributes to the improvement of network performance.

Firstly, without A2A, the model lacks local interactions within the prediction scenes, resulting in a decrease in model metrics.

Secondly, the absence of the temporal dependency module prevents the network from addressing temporal dependencies. Since inferring future trajectories of agents in highly dynamic environments heavily relies on historical information, the lack of the transformer encoder module significantly impairs the model’s performance metrics.

Thirdly, lane information plays a crucial role in motion prediction, as road environment information constrains the trajectories of vehicles to some extent. Under such constraints, vehicles generally move along the lanes. Moreover, global graph A2A also contributes to the model’s effectiveness, as global interactions can capture long-range dependency relationships, enhancing the accuracy of predictions.

#### 4.1.6. Qualitative Results of QINET

**Figure 4:**
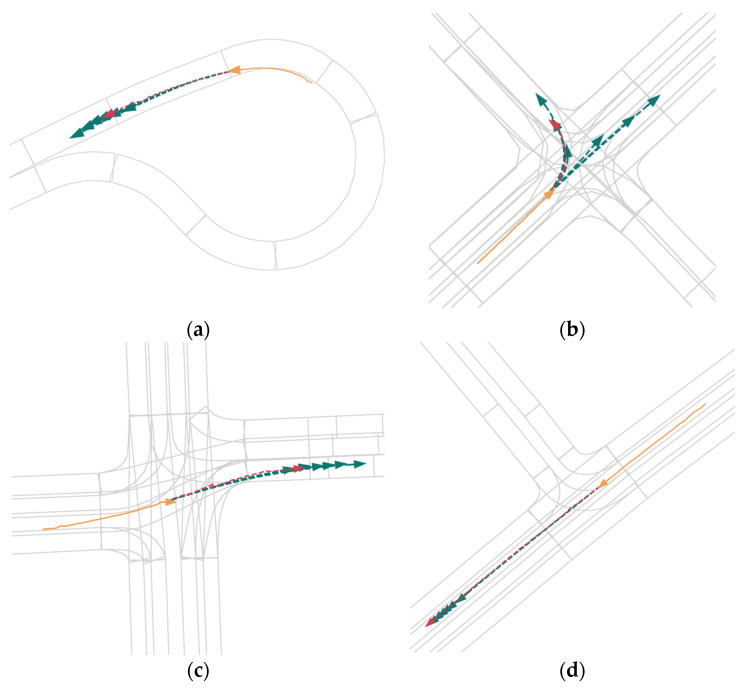
Qualitative Results of QINET. We selected several classical scenario prediction results as shown in (**a**–**d**). For clarity, we visualize individual agents separately. We use orange to depict past trajectories, red for actual trajectories, and green for predicted trajectories.

In the visualizations in Figure 4, we selected representative scenes to demonstrate the qualitative results of the QINET network. The visualizations confirm that QINET is capable of performing multimodal predictions for all agents, and the predicted trajectories are reasonable and close to the ground truth. For clarity, we display only the agent’s historical trajectory in yellow, the ground truth future trajectory in red, and the predicted trajectory in green. It can be observed that due to the presence of local A2A, temporal, L2A, and global A2A modules, our network effectively extracts agent features and predicts their future trajectories. Additionally, the query-informed multimodal scene encoding effectively promotes the multimodality of agent vehicle future trajectories.

#### 4.1.7. Comparison with HiVT in Bad Case

**Figure 5:**
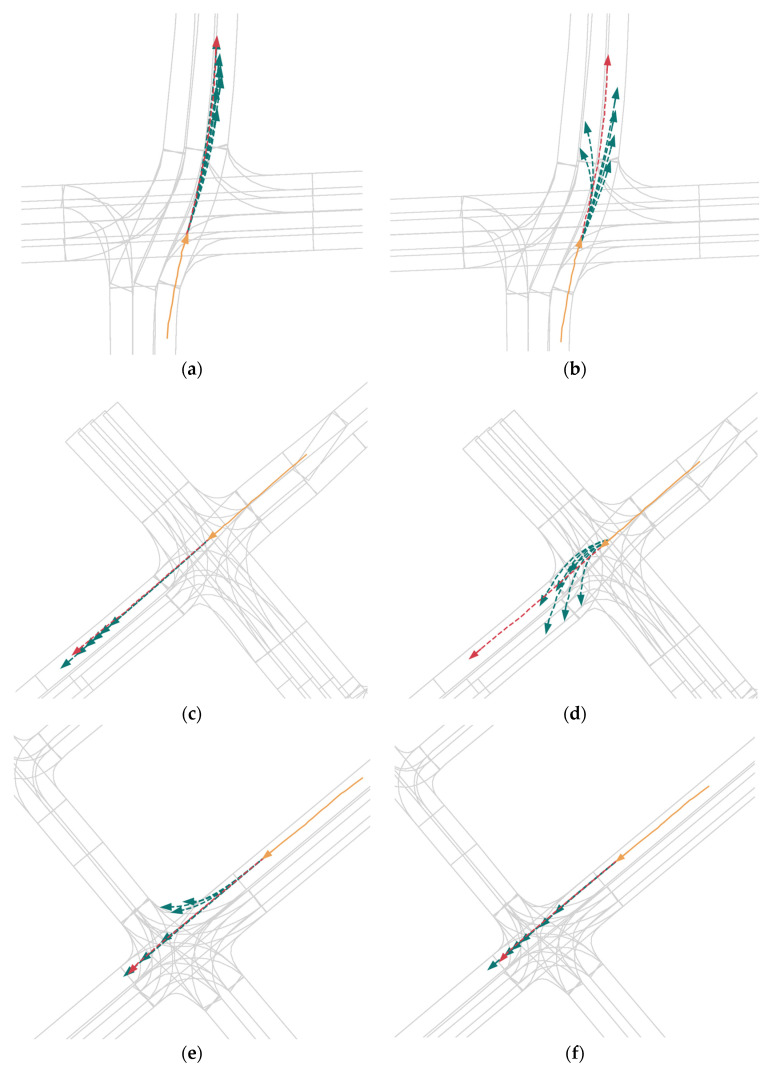
Visualize the comparative results. (**a**,**c**,**e**) come from QINET, while (**b**,**d**,**f**) come from HiVT.

In this part, we compared the output results of QINET with those of HiVT, as shown in the following Figure 5. It can be seen that some bad cases predicted in the HiVT model show better prediction results in the QINET model. In addition, in some intersection scenarios, the QINET network is capable of demonstrating awareness of turning. This is attributed to our decoder design that enhances the multimodality of the trajectory predictions.

#### 4.1.8. Failed Cases

**Figure 6:**
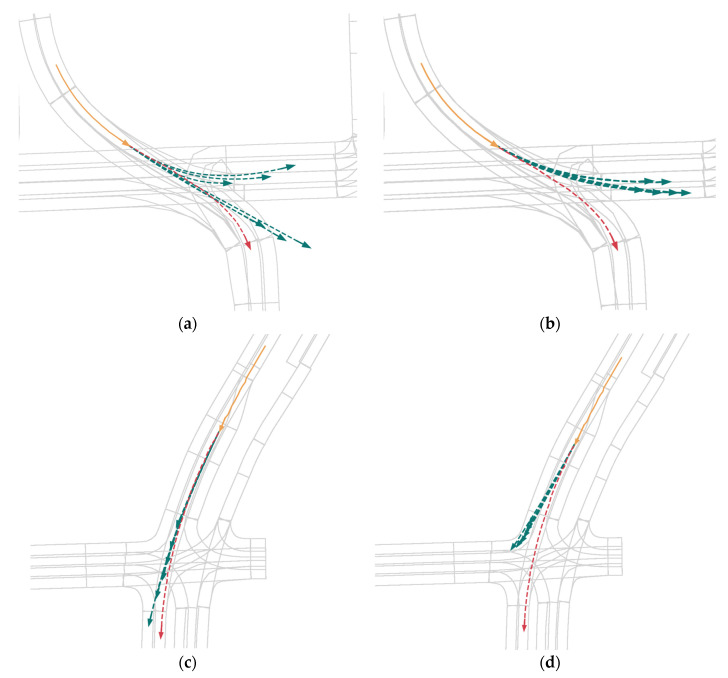
Visualize the comparative results. (**a**,**c**) come from QINET, while (**b**,**d**) come from HiVT.

In this section, we present some scenarios where QINET predictions failed, as shown in Figure 6. Compared to HiVT, QINET’s prediction results have improved, with trajectories becoming more multimodal. This improvement is due to the presence of multimodal scene query features in the decoder.

## 5. Conclusions

This paper presents a new multi-agent prediction framework that enhances trajectory prediction accuracy by constructing extended node features and edge features. It utilizes the query mechanism in cross-attention to obtain multi-scenario modal encodings, thereby maximally promoting the multimodality of generated trajectories. Experiments demonstrate that our method achieves good results in both prediction accuracy and the multimodality of generated trajectories on the Argoverse motion prediction benchmark. Future research will focus on how to conduct more efficient lane-to-agent (L2A) processing to improve the model’s inference speed. This is because considering all lane nodes within a certain radius can sometimes lead to resource wastage in some scenarios. For example, in scenarios where a vehicle is predicted to drive in the far-left lane, L2A would include nodes from the opposite lane, which may not be meaningful.

## Figures and Tables

**Table 1:** Results on Argoverse motion forecasting leaderboard.

Models	minFDE	minADE	MR
THOMAS [40]	1.4388	0.9423	0.1038
GOHOME [41]	1.4503	0.9425	0.1048
DenseTNT [26]	1.2815	0.8817	0.1258
mmTransformer [27]	1.3383	0.8436	0.1540
TNT [16]	1.4457	0.9097	0.1656
LaneRCNN [42]	1.4526	0.9038	0.1232
QINET	1.2643	0.8140	0.1436

**Table 2:** Comparison of QINET with HIVT on the Argoverse validation set without model ensembling.

Models (No Model Ensemble)	minFDE	minADE	MR
HiVT-128	0.6612	0.9691	0.0921
QINET	0.6514	0.9481	0.0892

**Table 3:** Importance of each component of our framework.

Extended A2A	Temporal Masked Attn	Scene Modality Creation	minADE	minFDE	MR
	✓	✓	0.6804	0.9843	0.0936
✓		✓	0.9628	1.4686	1.1977
✓	✓		0.6902	1.0376	0.1101
✓	✓	✓	0.6514	0.9481	0.0891

## Data Availability

The data presented in this study are available on request from the corresponding author.
